# Independent association of Lp(a) with platelet reactivity in subjects without statins or antiplatelet agents

**DOI:** 10.1038/s41598-022-21121-7

**Published:** 2022-10-05

**Authors:** Huixing Liu, Di Fu, Yonghong Luo, Daoquan Peng

**Affiliations:** Department of Cardiovascular Medicine, The Second Xiangya Hospital of Central South University, No. 139 Middle Renmin Road, Changsha, 410011 Hunan China

**Keywords:** Cardiology, Medical research, Risk factors

## Abstract

The physiological effect of Lp(a) on platelet activity is unclear. Previous studies explored the relationship between Lp(a) and platelet aggregation in patients taking statins and antiplatelet agents, but few was conducted in individuals without the bias of those drugs that either influence Lp(a) or platelet activity. The aim of this study was to assess the relationship between Lp(a) levels and platelet aggregation in subjects not taking statins or antiplatelet drugs. A hospital-based cross-sectional study was conducted to investigate the independent contribution of Lp(a) to platelet activity by controlling the effects of potential confounding factors including lipoprotein-associated phospholipase A2 [Lp-PLA2]. Blood samples were collected from 92 subjects without statins or antiplatelet agents from the Second Xiangya Hospital. The univariate correlation analysis showed a significant correlation between AA-induced average aggregation rate [AAR] and ApoB (r = 0.324, P = 0.002), ApoA1 (r = 0.252, P = 0.015), Lp(a) (r = 0.370, P < 0.001), Lp-PLA2 (r = 0.233, P = 0.025) and platelet counts [PLT] (r = 0.389, P < 0.001). Multivariate regression analysis suggested that Lp(a) contributed independently to AA-induced average aggregation rate (β = 0.023, P = 0.027) after controlling for the effects of ApoB, Lp-PLA2 and platelet counts. Lp(a) is positively associated with platelet aggregation independent of Lp-PLA2, which may partly account for the atherothrombotic effect of Lp(a).

## Introduction

Atherothrombotic disease is an important cause of morbidity and mortality. Platelet activation plays an important role in the pathological process of atherosclerosis and is involved in the whole process of thrombosis^1^. Prior clinical studies found a positive association between platelet activity and incident cardiovascular morbidity and mortality^2,3^. Platelet activity varies greatly among individuals, so exploring the factors that influence platelet activation is crucial for understanding atherothrombotic disease.

Lipoprotein(a) [Lp(a)] is a unique lipoprotein that has emerged as an independent risk factor for developing cardiovascular disease [CVD]. Lp(a) refers to lipoprotein that include apolipoprotein B100 [apoB100], oxidized phospholipid [OxPL] and apo(a). The level of Lp(a) is largely determined by gene and shows great variation in different populations and individuals^4,5^. Retrospective analyses suggest that reduced Lp(a) levels are associated with reduced cardiovascular risks^6^. The risk of myocardial infarction is evaluated at a cut-off for Lp(a) of 30–50 mg/dl^7^.

The pathogenic mechanisms of Lp(a)’s pro-thrombotic antifibrotic effects are widely recognized though the underlying mechanism has not been clearly revealed. On the one hand, sufficient evidence shows that apo(a) of Lp(a) influences the conversion of fibrinogen to fibrinolytic enzymes^8^, stimulating the increase of platelet reactivity^9,10^. On the other hand, recently insufficient evidence has shown that Lp(a) promotes thrombosis by stimulating platelet aggregation. In patients undergoing percutaneous coronary intervention [PCI] with dual antiplatelet therapy, a higher Lp(a) level were significantly correlated with a higher AA-induced platelet aggregation rate^11^. However, the results was confused by the fact that statins and antiplatelet drugs could increase Lp(a)^12^ and reduce the tendency of platelet aggregation^13^ respectively. The effect of Lp(a) on platelet aggregation has not been reported in population without statins and antiplatelet agents. Therefore, it is unknown the naive correlation between plasma Lp(a) and platelet aggregation.

In the present study, we revealed a positive correlation between plasma Lp(a) and platelet aggregation when stimulated by agonist arachidonic acid [AA] in subjects without statins and antiplatelet agents. This correlation was independent of the effect of ApoB, lipoprotein-associated phospholipase A2 [Lp-PLA2] and platelet counts. The results indicate that Lp(a) may promote platelet aggregation independent of Lp-PLA2 and provide new evidence and mechanisms for Lp(a)’s pro-atherogenic effects.

## Results

### Study population

Baseline clinical characteristics and laboratory tests of participants according to the median of Lp(a) are summarized in Table 1. The study subjects were composed of 55 male and 37 female with an average age of 55 (± 12) years old. Total cholesterol [TC], low-density lipoprotein cholesterol [LDL-C], apolipoprotein B [ApoB], apoA1, nonesterified fatty acid [NEFA], Lp-PLA2 and platelet counts [PLT] were significantly higher in higher Lp(a) patients compared to the lower group. Other parameters, including gender, hypertension, diabetes and smoking, had no statistically significant differences between two groups.

**Table 1:** Characteristics of population.

	Total (n = 92)	Lower Lp(a) (61.1 [35.4, 79.8] mg/L) (n = 46)	Higher Lp(a) (180.4 [124.1, 274.7] mg/L) (n = 46)	P-value
Age, years	55 ± 12	54 ± 13	56 ± 11	0.450
Male, n (%)	55 (60)	29 (63)	26 (57)	0.526
Hypertension, n (%)	18 (20)	10 (22)	8 (17)	0.601
Diabetes, n (%)	11 (12)	6 (13)	5 (11)	0.749
Smoking, n (%)	26 (28)	13 (28)	13 (28)	1.000
TC, mmol/L	3.67 ± 0.89	3.42 ± 0.80	3.91 ± 0.92	0.007**
TG, mmol/L	0.97 (0.74, 1.36)	0.91 (0.71, 1.26)	0.98 (0.89, 1.41)	0.072
HDL-C, mmol/L	0.96 ± 0.28	0.95 ± 0.29	0.98 ± 0.27	0.616
LDL-C, mmol/L	2.32 ± 0.72	2.10 ± 0.61	2.54 ± 0.76	0.003**
ApoA1, g/L	0.79 ± 0.21	0.77 ± 0.22	0.69 ± 0.18	0.296
ApoB, g/L	0.63 ± 0.19	0.56 ± 0.18	0.69 ± 0.18	0.001**
NEFA, mmol/L	0.37 (0.26, 0.51)	0.33 (0.24, 0.41)	0.45 (0.32, 0.57)	0.001**
Lp-PLA2, U/L	320.7 ± 109.6	290.6 ± 97.8	350.9 ± 113.5	0.008**
PLT, × 10^12^/L	220 ± 81	196 ± 69	243 ± 85	0.005**

*Lp*(*a*) lipoprotein a, *TC* total cholesterol, *TG* triglyceride, *HDL-C* high density lipoprotein-cholesterol, *LDL-C* low density lipoprotein-cholesterol, *ApoA1* apolipoprotein A1, *ApoB* apolipoprotein B, *NEFA* nonesterified fatty acid, *Lp-PLA2* lipoprotein-associated phospholipase A2, *PLT* platelet counts.

### Factors correlate with AA-induced platelet reactivity

**Figure 1:**
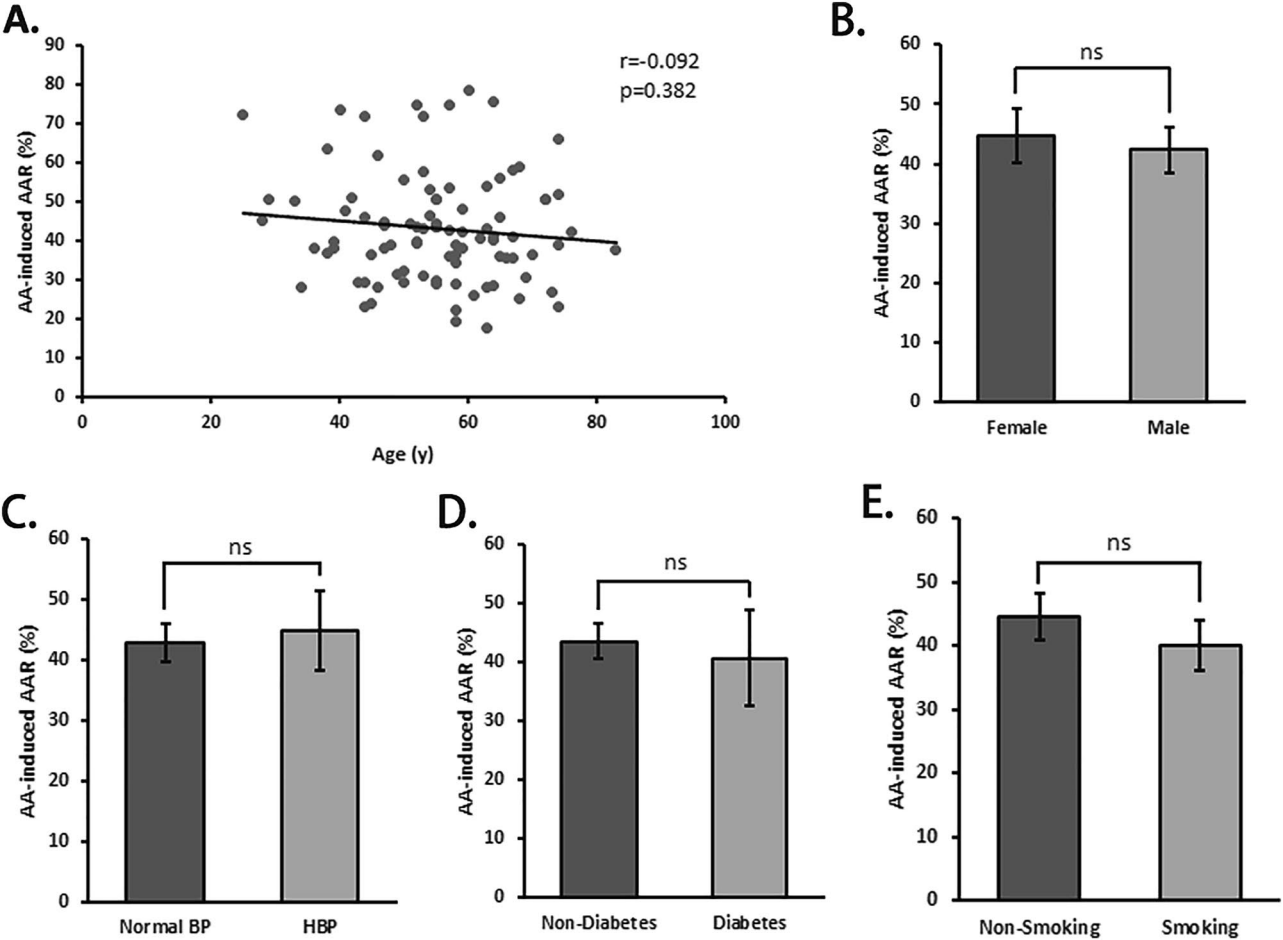
AA-induced platelet aggregation stratified by characteristic. AA-induced platelet average aggregation rate (AAR) in healthy subjects stratified by age (**A**), gender (**B**), hypertension (**C**), smoking (**D**), diabetes (**E**).

**Figure 2:**
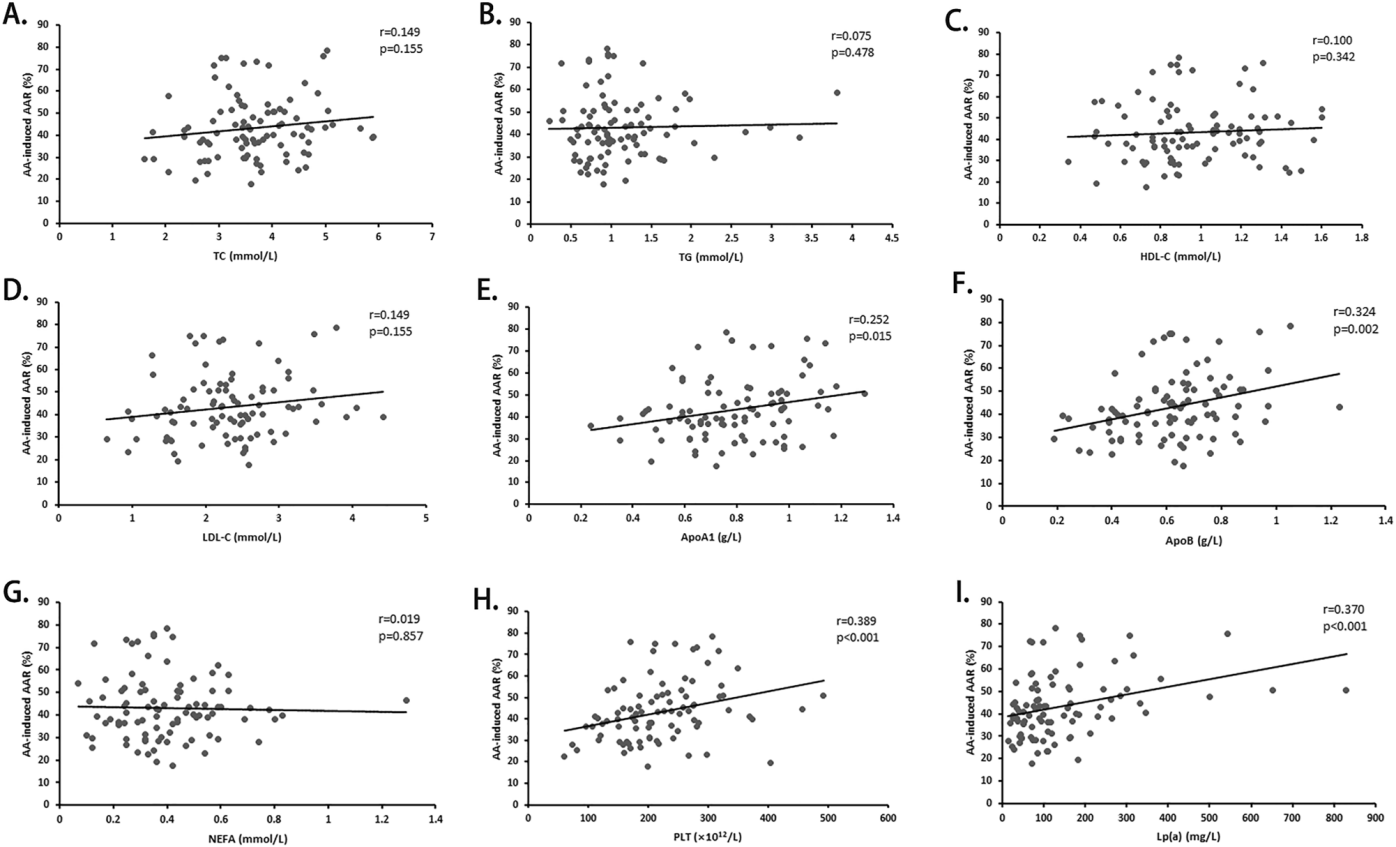
Correlation analysis of AA-induced platelet aggregation. Univariate linear correlation of TC (**A**), TG (**B**), HDL-C (**C**), LDL-C (**D**), ApoA1 (**E**), ApoB (**F**), NEFA (**G**), PLT (**H**), and Lp(a) (**I**) with AA-induced platelet average aggregation rate (AAR).

No significant differences of AA-induced AAR were observed in subgroups according to the status of gender, hypertension, diabetes and smoking (Fig. 1). Correlation analysis revealed significant correlations of AA-induced AAR with ApoA1 (r = 0.252, P = 0.015) (Fig. 2E), ApoB (r = 0.324, P = 0.002) (Fig. 2F) and PLT (r = 0.389, P < 0.001) (Fig. 2H) instead of other Blood lipid indices (Fig. 2A–D,G).

In accordance with correlation analysis, a significant higher AA-induced AAR was observed in subjects with higher ApoA1 level (P = 0.042), higher ApoB groups (P = 0.037) and higher PLT groups (P < 0.001). Also, subjects with AA-induced AAR higher than median value had significant higher ApoA1 level (P = 0.005), higher ApoB levels (P = 0.001) and higher PLT (P < 0.001).

### Plasma Lp(a) concentration and platelet reactivity

A direct linear correlation was found between increased plasma Lp(a) levels and AA-induced AAR (r = 0.370, P < 0.001) (Fig. 2I). When assessed according to median value of Lp(a), there was a significant increase in AA-induced AAR in the higher Lp(a) group compared to the lower one (P = 0.009). On the other hand, subjects with high AA-induced AAR had significant higher serum level of Lp(a) (P = 0.003). After controlling LDL-C, ApoA1, ApoB, PLT and Lp-PLA2, AA-induced AAR was still inversely correlated with Lp(a) concentrations.

**Figure 3:**
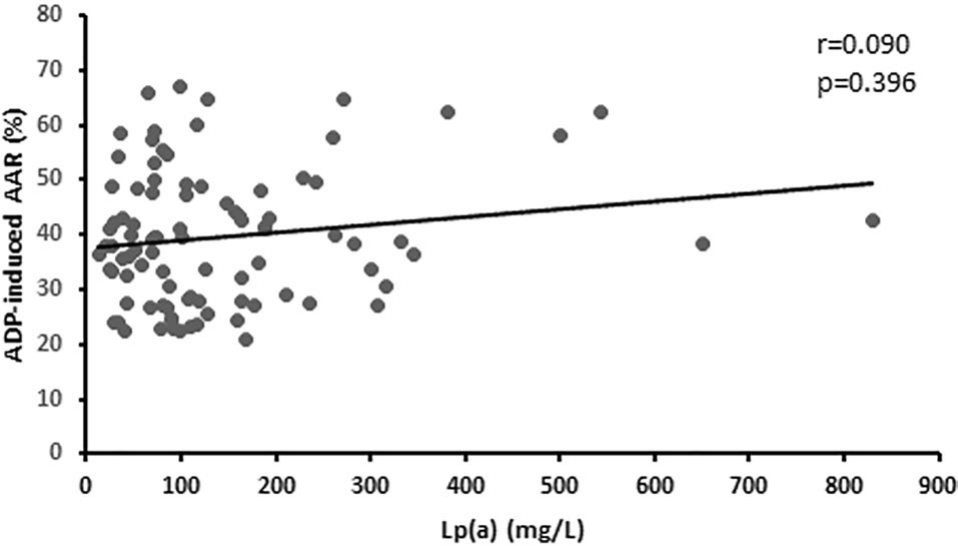
Association between Lp(a) levels and ADP-induced platelet average aggregation rate in subjects. Univariate linear correlation analysis of serum Lp(a) levels with ADP-induced platelet average aggregation rate (AAR).

In order to explore the relationship between Lp(a) and ADP-induced platelet aggregation, we performed a correlation analysis. However no direct linear correlation was found between increased plasma Lp(a) levels and ADP-induced AAR. (r = 0.090, P = 0.396) (Fig. 3).

### Lp-PLA2 activity and plasma Lp(a) concentration/AA-induced platelet reactivity

**Figure 4:**
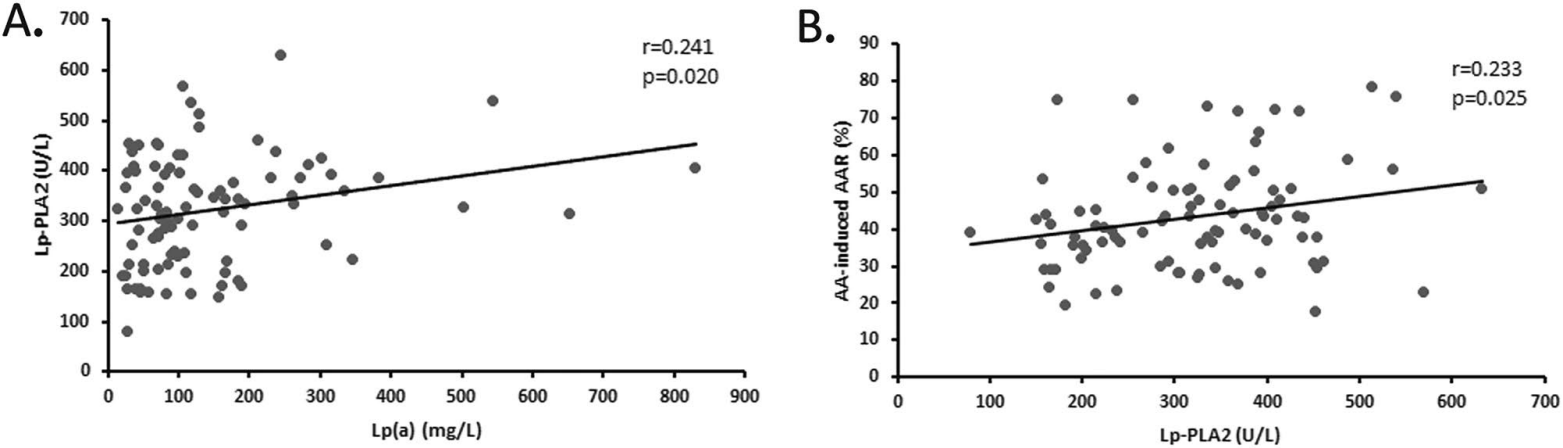
Association between plasma Lp-PLA2 and Lp(a) levels/AA-induced platelet average aggregation rate in subjects. (**A**) Univariate linear correlation analysis of serum Lp(a) levels with Lp-PLA2 activities. (**B**) Univariate linear correlation analysis of the serum Lp-PLA2 activities with AA-induced platelet average aggregation rate (AAR).

A direct linear correlation was found between increased plasma Lp(a) levels and Lp-PLA2 activity (r = 0.241, P = 0.020) (Fig. 4A). Subjects with higher Lp-PLA2 levels had significant higher serum levels of Lp(a) (P = 0.006)Also, increased plasma Lp-PLA2 activity was correlated increased AA-induced AAR (r = 0.233, P = 0.025) (Fig. 4B).

### Multivariate linear regression analysis

In the multivariate regression analysis, all related factors including Lp(a), Lp-PLA2, ApoA1, ApoB and PLT were included in model 1 with Lp(a) (β = 0.021, P < 0.005) and PLT (β = 0.041, P = 0.034) and there is no multicollinearity among these factors. Model 2 is a stepwise multivariate regression model and only parameters of covariates that were retained in the model during stepwise elimination procedure are included in the table. Lp(a) (β = 0.023, P = 0.027), ApoB (β = 18.242, P = 0.016) and PLT (β = 0.040, P = 0.023) were found to predict AA-induced AAR. The adjusted R^2^ of the multivariate model was 0.184, P < 0.001 (Table 2).

**Table 2:** The results of the multivariate linear regression analysis regarding the association of AA-induced AAR and characteristics.

Variables	Model 1 (adjusted R^2^ 0.189, p < 0.001)			Model 2 (adjusted R^2^ 0.194, p < 0.001)		
	β	Standardized β	P-value	β	Standardized β	P-value
Lp(a)	0.021	0.204	< 0.050	0.023	0.226	0.027
Lp-PLA2	− 0.004	− 0.029	0.823	–	–	–
ApoA1	8.855	0.129	0.218	–	–	–
ApoB	16.776	0.221	0.088	18.242	0.240	0.016
PLT	0.041	0.234	0.020	0.040	0.227	0.023

*AA* arachidonic acid, *AAR* average aggregation rate, *Lp*(*a*) lipoprotein a, *Lp-PLA2* lipoprotein-associated phospholipase A2, *ApoA1* apolipoprotein A1, *ApoB* apolipoprotein B, *PLT* platelet counts.

### Hierarchical multiple regression analysis

In this study, hierarchical multiple regression was used to predict the effect of Lp(a) on AA-induced AAR after adjustment for PLT or ApoB. Lp(a) explained 11.1% of the AA-induced AAR variation (adjusted R^2^ = 10.1%, F = 11.269, P = 0.001) while PLT (model 1) explained 9.4% (adjusted R^2^ = 8.4%, F = 9.133, P = 0.003) and ApoB (model 3) explained 10.1% (adjusted R^2^ = 9.1%, F = 10.158, P = 0.002). After adjustment for PLT (model 2) and ApoB (model 4) respectively, Lp(a) explained 7.2% (ΔF = 7.541, P = 0.007) and 7.1% (ΔF = 7.583, P = 0.007) of the AA-induced AAR variance (Table 3).

**Table 3:** The results of the hierarchical multiple regression analysis regarding the association of AA-induced AAR and characteristics.

	β	AA-induced AAR					
		Model 1	Model 2	Model 3	Model 4	Model 5	Model 6
Step 1	PLT	0.054	0.042	–	–	0.048	0.040
	ApoB	–	–	23.461	18.682	21.717	18.242
Step 2	Lp(a)	–	0.028	–	0.028	–	0.023
	F	9.133	8.676	10.158	9.242	9.225	8.129
	ΔF	9.133**	7.541**	10.158**	7.583**	9.225**	5.075*
	R^2^	0.094	0.166	0.101	0.172	0.175	0.221
	ΔR^2^	0.094**	0.072**	0.101**	0.071**	0.175**	0.046*

*P < 0.05, **P < 0.01.

*AA* arachidonic acid, *AAR* average aggregation rate, *Lp*(*a*) lipoprotein a, *ApoB* apolipoprotein B, *PLT* platelet counts.

### Mediation analysis

**Figure 5:**
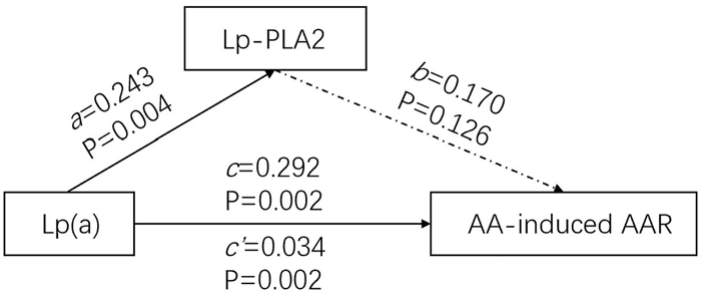
Mediation models of Lp-PLA2 in Lp(a) and AA-induced AAR. The direct effect of Lp(a) on AA-induced AAR (*c*) was significant and estimated to be 0.292 with *p* = 0.126. The direct effect of Lp(a) on Lp-PLA2 (*a*) was significant and estimated to be 0.243 with *p* = 0.004. The direct effect of Lp-PLA2 on AA-induced AAR (*b*) was insignificant and estimated to be 0.170 with *p* = 0.126. The total effect of Lp(a) on AA-induced AAR (*c*′ = *c* + *ab*) was significant and estimated to be 0.034 with *p* = 0.002.

The effects of the model that Lp(a) has effect on AA-induced AAR through Lp-PLA2 (Table 4) show that the total effect (*c*′) is 0.034 and the direct effect (*c*) is 0.030. When the mediator variable (Lp-PLA2) was added to the model, no statistically significant mediating effect was found (*ab* = 0.004, P = 0.093). The value of 0.243 for the *b* path between Lp-PLA2 and AA-induced AAR is insignificant (P = 0.126) (Fig. 5).

**Table 4:** Total, direct and indirect effects of the median model that Lp(a) has effect on AA-induced AAR through Lp-PLA2.

Effect	Point estimate	Product of coefficients		Bootstrapping				*P* value
				Bias-correlated 95% CI		Percentile 95% CI		
		SE	Z	Lower	Upper	Lower	Upper	
Total effect (*c*′)	0.034	0.011	3.091	0.018	0.060	0.018	0.062	0.002
Direct effect (*c*)	0.030	0.012	2.500	0.012	0.057	0.014	0.059	0.002
Indirect effect (*ab*)	0.004	0.004	1.000	− 0.001	0.015	− 0.001	0.013	0.093

*c*′ total path of the whole model, *c* direct effect of Lp(a) on AA-induced-AAR, *ab* product of coefficients, *Lp*(*a*) lipoprotein a, *AA* arachidonic acid, *AAR* average aggregation rate, *Lp-PLA2* lipoprotein-associated phospholipase A2.

## Discussion

Previous studies have shown that multiple genetic and non-genetic factors, such as age, gender and white blood cell count, have an effect on platelet function^14^, but fewer articles have examined the effect of plasma Lp(a) levels on platelet aggregation. We found out that Lp(a) variation accounted for 11.1% of platelet aggregation while platelet count only accounted for 9.4% and ApoB accounted for 10.1%; Lp(a) variation still explained 7.2% or 7.1% of platelet aggregation after adjustment for platelet count or ApoB respectively. Our results confirm an independent positive correlation between Lp(a) levels and platelet aggregation independent of ApoB, Lp-PLA2 and platelet counts in a population without statin and antiplatelet agents for the first time.

Platelet activation is a key process in both protective hemostasis and pathological thrombosis through the activation of multiple pathways by the binding of several agonists, like adenosine diphosphate [ADP] and arachidonic acid [AA]. These agonists and intracellular signals activate the integrin receptor GPIIb/IIIa on the platelet surface, which produces stronger adhesion between platelets^15^. Then platelet further releases more ADP which could activate receptors P2Y_12_, P2Y_1_ on the platelet surface membrane. AA contained in platelet was converted to thromboxane A2 [TXA2], which binds to thromboxane receptors on the platelet surface membrane and promotes intraplatelet calcium release^16^. High intraplatelet calcium leads to enhanced procoagulant effects^17^, ultimately leading to irreversible platelet aggregation.

Platelet reactivity refers to the degree of the response of blood platelet to an external stimulus. ADP and AA are often added in blood samples to test platelet reactivity. Light transmission aggregometry (LTA) is considered as the “gold standard” to measure platelet aggregation in platelet-rich plasma. However, it is complex and technically demanding. Impedance aggregometry, which is based on the principle of electrical impedance, is a newer approach to measuring platelet aggregation. It could measure platelet aggregation in whole blood samples by amplifying and recording the small current or impedance changes between electrode probes. Therefore, it is more physiological than studies performed in platelet-rich plasma^18^.

There are many factors that are positively associated with platelet aggregation, such as ApoB^19^ and platelet count^20^. The same results were obtained in our experiment. Although most researches showed that higher LDL-C concentrations increase platelet activity^21^, a Japanese cross-sectional study did not find a positive association between LDL-C and platelet activity as shown in our study^22^. A possible reason is the complex action of lipids on platelets in the body. For example, naturally oxidized LDL can inhibit platelet aggregation^23–25^, which could partly explain the inconsistent results between in vivo and in vitro experiments.

However, there are no clinical studies exploring the relationship between Lp(a) and platelet aggregation rate excluding the influence of statins and antiplatelet agents. In our experiment, we found that Lp(a) was associated with AA-induced platelet aggregation in a multivariate regression model independent of ApoB and platelet counts. In this model, there is no collinearity among Lp(a) and ApoB, perhaps because ApoB not only exists in Lp(a), but also in LDL, very low-density lipoprotein [VLDL] and chylomicron [CM].

Similar to our findings, Zhu et al. found that in patients undergoing PCI with dual antiplatelet therapy, those with higher serum Lp(a) levels had higher AA-induced platelet aggregation^11^. Other in vitro studies have also shown that Lp(a) can increase AA-induced platelet aggregation^26,27^. The possible mechanisms may be that apo(a) can enhance platelets’ responses to the thrombin receptor-activating peptide SFLLRN^27^ and that the OxPL on Lp(a) can promote platelet activation by interacting with the CD36 (platelet glycoprotein IV) receptor on platelets^28^.

A large number of studies have found positive correlations between Lp(a) and both Lp-PLA2 concentration and activity similar to our study^29–31^. Lp-PLA2 on Lp(a) can bind to and hydrolyze OxPL to produce pro-inflammatory and pro-apoptotic lipid regulators^32^. Lp-PLA2 is also known as platelet-activating factor-acetohydrolase [PAF-AH], which could inactivate PAF (platelet Agonist). We therefore speculate that Lp-PLA2 may affect platelet aggregation.

In our experiment, we found Lp-PLA2 is positively associated with AA-induced platelet aggregation. However, Lp(a) contributed independently to AA-induced AAR after controlling for the effect of Lp-PLA2 and Lp-PLA2 is not a mediator of Lp(a) affecting platelet aggregation. The result demonstrates that the effect of Lp(a) on platelet aggregation was not dependent on Lp-PLA2, which is corroborated by the results of an in vitro experiment^33,34^. Platelet agonist PAF in vivo is majorly cleared from endothelium-rich organs (e.g., liver) rather than Lp-PLA2 activity^35^, partly explain that Lp(a) may not influence platelet aggregation through Lp-PLA2. Another possible reason is that apo(a) may reduce the catalytic efficacy of Lp-PLA2 on OxPL^36^, the effect of Lp(a) on platelet aggregation is influenced by both apo(a) and OxPL.

We didn’t find any correlation between Lp(a) and ADP- induced platelet aggregation. This result is supported by findings that elevated Lp(a) concentrations did not lead to altered ADP-induced platelet aggregation in vitro studies^27,37^. Similar to our findings, Salsoso et al. found that in patients with and without aspirin and statin therapy, serum Lp(a) levels was not associated with ADP-induced platelet aggregation^38^. But the opposite results showed that higher serum Lp(a) levels had higher ADP-induced platelet aggregation in patients with dual antiplatelet therapy^11^. The inconsistency of the results may be confounded by the influence of antiplatelet agents. Some researchers found that apo(a) can reduce ADP-induced platelet reactivity^39,40^ by increasing intracellular cyclic adenosine monophosphate (cAMP)^41^, which may cancel out the promoting effect of Lp(a) on platelet aggregation.

There are still some limitations in this study. Firstly, this study is a cross-sectional study with a relatively small study sample size. Therefore, the conclusions can only suggest correlation and cannot determine causality. Secondly, this study only reported a phenomenon and did not explore the mechanism of Lp(a) action on platelet function. All of these deserve further attention.

## Conclusions

In conclusion, Lp(a) levels were positively associated with platelet aggregation independent of Lp-PLA2 in population without statins and antiplatelet agents, providing new evidence for Lp(a)’s pro-atherogenic effects. The underlying mechanisms deserve further investigation.

## Methods

### Study population and design

This study is a cross-sectional, single center clinical study. 92 subjects with bone fracture, urinary systematic calculi and gastritis were recruited from the Second Xiangya Hospital of Central South University, Changsha, China. To investigate the relationship between Lp(a), Lp-PLA2 and platelet aggregation rate, we studied all relevant indexes of all the subjects. Exclusion criteria included: coronary heart disease, stroke, peripheral vascular disease, heart failure, acute coronary syndrome, renal failure, chronic liver disease, hyperthermia or bacterial/viral infection, autoimmune disease, arthritis, malignancy, severe diabetes mellitus, hypertension and other serious medical diseases. All the subjects didn’t take any statin and antiplatelet agent. The study was approved by the Medical Ethics Committee of the Second Xiangya Hospital of Central South University and was conducted in accordance with the approved guidelines and regulations. Informed consent was obtained from all subjects and/or their legal guardian(s).

### Blood lipids measurements

A peripheral blood sample was collected from the patient's arm vein. Subjects fasted for at least 10 h prior to blood collection and blood was assessed by Japanese HITACHI 7600 fully automatic biochemical analyzer (HITACHI, Japan) and its supporting reagents for routine blood and lipid parameters, including total cholesterol [TC], triglycerides [TG], low-density lipoprotein cholesterol [LDL-C], high-density lipoprotein cholesterol [HDL-C], apolipoprotein A1 [ApoA1], apolipoprotein B [ApoB], nonesterified fatty acid [NEFA] and lipoprotein(a) [Lp(a)].

### Assessment of platelet aggregation

A blood sample was withdrawn after an overnight fasting and analyzed for platelet aggregation within 2 h. Whole blood aggregation was determined using PL-11 platelet analyzer (SINNOWA, Nanjing). The system detects the electrical impedance change due to the adhesion and aggregation of platelet on two independent electrode-set surfaces. Sodium citrate was used as anticoagulant, adenosine diphosphate and arachidonic acid as agonists. A 1:9 dilution of whole blood anticoagulated with sodium citrate and 0.9% NaCl was stirred at 25 °C. ADP 50 μmol/L or AA 2 mg/mL were added.

### Assessment of Lp-PLA2 activity

The lipoprotein-associated phospholipase A2 [Lp-PLA2] activity in serum was measured by a Japanese HITACHI 7600 fully automated biochemical analyzer (HITACHI, Japan). The assay method was continuous monitoring. The kit and supporting calibrators and quality control products were provided by Shanghai DiaSys diagnostic systems GmbH.

### Statistical analysis

Statistical analyses were performed with the Statistical Package for the Social Sciences, version 25.0, and clinical data were expressed as mean ± standard deviation (continuous data with normal distribution) or median of interquartile range (continuous data with skewed distribution). Comparisons between categorical data were performed with Chi Squared tests, while continuous variables were assessed by unpaired *t* test (for normal distribution) or nonparametric test (for skewed distribution). To assess the correlation between variables, Spearman correlation analysis was used. Stepwise multiple linear regression analysis and hierarchical multiple regression analysis was used to identify independent variables that were significantly associated with platelet aggregation rate, including all potential variables with significant relationships. In correlation and regression analyses, normalized transformation was used for variables with skewed distribution. Two-tailed P values < 0.05 were considered statistically significant. Mediating effects of Lp-PLA2 in Lp(a) and AA-induced AAR using the AMOS 24.0 model. The *c* path is the direct effect of treatment on outcome, before taking into account the effects of specific mediating variables. Paths *a* and *b* make up the mediating pathway, with the mediating effect usually being described in the literature as the product of coefficients (*ab*)^42^. The *c*′ path denotes the total effect of the whole model (*ab* + *c*).

## Data Availability

The datasets generated and/or analyzed during the current study are not publicly available due to restrictions according to patient privacy regulations but are available from the corresponding author on reasonable request.
